# The MIT domain of chitin synthase 1 from the oomycete *Saprolegnia monoica* interacts specifically with phosphatidic acid

**DOI:** 10.1016/j.bbrep.2022.101229

**Published:** 2022-02-10

**Authors:** Christian Brown, Joan Patrick, Jobst Liebau, Lena Mäler

**Affiliations:** Department of Biochemistry and Biophysics, Stockholm University, SE-106 91, Stockholm, Sweden

## Abstract

Chitin synthases are vital for growth in certain oomycetes as chitin is an essential component in the cell wall of these species. In *Saprolegnia monoica*, two chitin synthases have been found, and both contain a Microtubule Interacting and Trafficking (MIT) domain. The MIT domain has been implicated in lipid interaction, which in turn may be of significance for targeting of chitin synthases to the plasma membrane. In this work we have investigated the lipid interacting properties of the MIT domain from chitin synthase 1 in *Saprolegnia monoica*. We show by fluorescence spectroscopy techniques that the MIT domain interacts preferentially with phosphatidic acid (PA), while it does not interact with phosphatidylglycerol (PG) or phosphatidylcholine (PC). These results strongly suggest that the specific properties of PA are required for membrane interaction of the MIT domain. PA is negatively charged, binds basic side chains with high affinity and its small headgroup gives rise to membrane packing defects that enable intercalation of hydrophobic amino acids. We propose a mode of lipid interaction that involves a combination of basic amino acid residues and Trp residues that anchor the MIT domain specifically to bilayers that contain PA.

## Highlights

• MIT from chitin synthase 1 in *S. monoica* interacts specifically with PA.
• Interactions with lipids is does not depend only on charge.
• MIT does not alter its structure in response to lipids.
• Specific interaction with PA suggests a role for MIT in trafficking or insertion of chitin synthase 1.

## Graphical abstract

**Figure:**
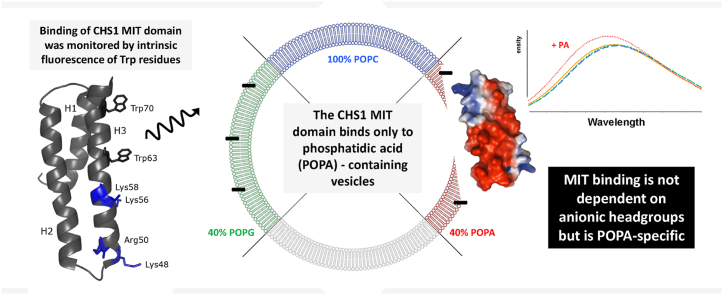

## Abbreviations

CD: circular dichroism
CHS: chitin synthase
CPE: ceramide phospatidylethanolamine
MIT domain: Microtubule Interacting and Trafficking domain
MMD: Myosin motor-like domain
MMD PA: phosphatidic acid
PAGE: polyacrylamide gel electrophoresis
PC: phosphatidylcholine
PE: phosphatidylethanolamine
PG: phosphatidylglycerol
POPA: 1-palmitoyl-2-oleoyl-sn-glycero-3-phosphatidic acid
POPC: 1-palmitoyl-2-oleoyl-sn-glycero-3-phosphatidylcholine;
PC: phosphatidylcholine
POPE: 1-palmitoyl-2-oleoyl-sn-glycero-3-phosphatidylethanolamine
POPG: 1-palmitoyl-2-oleoyl-sn-glycero-3-phosphatidylcholine;
PS: phosphatidylserine
SEC: size exclusion chromatography
SmCHS: *Saprolegnia monoica* chitin synthase

## 1 Introduction

Oomycetes are eukaryotic microorganisms that are similar to but evolutionarily distinct from fungi [1]. A significant number of oomycetes are pathogens of plants and animals and cause infections that create significant economic losses for e.g. the agricultural and fishing industry [2,3]. A potential approach to combating oomycete pathogens is to target enzymes involved in cell wall biosynthesis. The cell walls of oomycetes contain cellulose, together with β-(1 → 3)- and β-(1 → 6)-glucans. In addition, the cell walls of some oomycetes, such as *Saprolegnia monoica*, also contain a small amount of chitin, which is synthesized by chitin synthases (CHS) [4,5]. CHS are integral membrane proteins and belong to the glycosyltransferase family 2 (GT2) [6]. Although chitin is present only in small amounts, it has been demonstrated to be vital for survival [7] and specific chitin synthase inhibitors have been shown to disrupt the cell wall and cause lysis as well as bursting of hyphal tips [7]. Moreover, it has been demonstrated that CHS are present in detergent-resistant domains of the *Saprolegnia monoica* plasma membrane [8], supporting the notion that CHS localizes to the apex of hyphae.

Little is known about CHS transport in cells. In fungi, small spheroidal vesicles called chitosomes have been isolated in different species and have been shown to transport CHS to the growth tip region and to synthesize chitin *in vitro* [9,10]. However, in oomycetes such vesicles specialized in transport of CHS have not been found [11]. Some CHS from filamentous and dimorphic fungi contain a myosin motor-like domain (MMD) at the N-terminus. Studies suggest that MMD may assist the transport of CHS to the hyphal tip via interactions with microtubules and/or actin [[12], [13], [14]]. Thus, chitin synthases are potentially interesting targets to inhibit pathogen growth. Since CHS are most likely to be found in the apex of the hyphae, a better understanding of their delivery to and insertion into the hyphal plasma membrane could help to develop new strategies to inhibit the growth of such a pathogen.

**Fig. 1:**
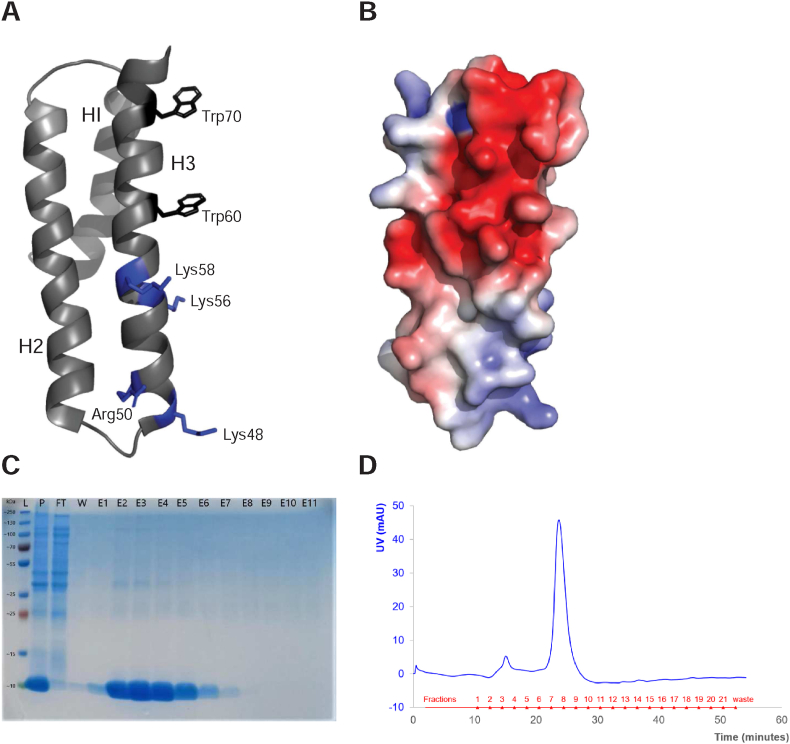
Structure and purity of the MIT domain (PDB accession code 2MPK) in *S. monoica* chitin synthase 1. A: Structure of the MIT domain shown as ribbon diagram with the three helices numbered and in which the location of the two intrinsic Trp residues and the basic residues in helix 3 (coloured in blue) are indicated. B: Surface charge representation of the MIT domain with blue colour indicating cationic and red colour indicating anionic surface charge. C: SDS-PAGE gel indicating purity of the MIT domain as eluted from IMAC column. L = PageRuler Plus Prestained Protein Ladder (Thermo Scientific); P = pre-lysis resuspended cell pellet; FT = IMAC flow-through; W = IMAC wash; E1 – E11 = 1 ml IMAC elutions. D: SEC profile of MIT domain. (For interpretation of the references to colour in this figure legend, the reader is referred to the Web version of this article.)

In *S. monoica* two putative chitin synthase (Chs) genes (SmChs 1 and SmChs 2) were previously identified [15]. Work by Guerriero et al. further identified the presence of a Microtubule Interacting and Trafficking (MIT) domain at the N-terminus of both CHS 1 and 2 [7]. MIT domains are small and typically contain three helices arranged in an antiparallel fashion [[16], [17], [18]]. They bind to MIT-interacting motifs [19,20], but direct lipid interactions may also be of importance for the role of MIT domains in protein trafficking and localization [18,21]. It was demonstrated *in vitro* that the MIT domain from CHS 1 has affinity for certain negatively charged lipids, most notably phosphatidic acid (PA) [18]. These results led to the proposal that the MIT domain could be involved in the trafficking, and/or recycling of CHS enzymes in *S. monoica*. MD simulations suggested that the lipid interaction occurs mainly through a lipid-interacting “hot-spot” that involves Arg residues in helices 1 and 2 [22] (see Fig. 1), although several alternative interaction modes were also suggested. For instance, the N-terminal part of helix 3 contains four basic residues that could be implicated in lipid binding, while the C-terminus contains more acidic residues (Fig. 1).

Phosphatidic acid (PA) is present in the plasma membrane of *S. monoica* [8] but the main components are phosphatidylcholine (PC), phosphatidylethanolamine (PE) and ceramide-phosphatidylethanolamine (CPE), with minor amounts of phosphatidylserine (PS) and phosphatidylinositol (PI). The lipids contain a high proportion of unsaturated fatty acyl chains. Although PA is only present in low overall amounts in the cell wall, the local proportions of PA in different membrane regions of *S. monoica* are not well known, yet it is known to have important functions in membrane dynamics [23] and cell signaling [24]. PA has a propensity to induce membrane curvature stress through alteration of the lipid packing in the bilayer, which facilitates protein insertion in the cell membrane [25] and some proteins display a high affinity specifically for PA [26,27]. The suggested mechanism by which this is achieved has been termed "electrostatic/hydrogen bond switch model" [28]. In this model Lys in particular (but also Arg to a lesser extent) come into proximity of PA via electrostatic interactions. Once the approach is close enough, a hydrogen bond is formed to the phosphate headgroup of PA and its charge decreases from −1 to −2 enhancing electrostatic interactions. The binding is further enhanced by the negative curvature stress of PA. This allows hydrophobic residues to form hydrophobic interactions with the acyl chains which further stabilizes the interactions. It is thus the combination of strong electrostatic interactions and hydrophobic interactions facilitated by PA's small head group that give rise to the specific affinity of some proteins for PA, as no other lipid has this combination of properties [28]. No consensus PA binding motif has been identified but PA binding domains are enriched in Lys, Arg, His, Ser and Trp residues [26,27].

In this work, we have characterized the interactions between the MIT domain of SmCHS 1 and phospholipids using a variety of biophysical methods. Vesicles containing different lipid compositions were used to test if MIT interacts specifically with lipids, including PA, PE, PC and PG. The latter lipids were used as generic models for zwitterionic and negatively charged headgroups, while PE is abundant in the plasma membrane and is also a non-bilayer forming lipid. Our results demonstrate that the MIT domain interacts with PA, but not with PC or PG to the same extent. No evidence for structural changes in the MIT domain was observed upon interaction. The affinity of the MIT domain for PA substantiates the hypothesis of a specific role of the MIT domain in trafficking or recycling of CHS.

## 2 Materials & methods

### 2.1 Protein expression and purification of the MIT domain in *E. coli*

Protein expression and purification were performed as previously described in Brown et al., 2016 [18]. However, size exclusion chromatography performed on an ÄKTA purifier FPLC (GE Healthcare, Uppsala, Sweden) was added as an extra step of purification after affinity chromatography. The purity of the protein was assessed by SDS PAGE analysis (Mini-PROTEAN TGX, Bio-rad, USA). Elution samples were pooled and concentrated using 3 kDa cut-off Amicon Ultra centrifugal filters (Millipore, Cork, Ireland) prior to being loaded onto a Superose 12 column (GE Healthcare, Uppsala, Sweden) and the MIT domain was eluted in 50 mM phosphate buffer pH 7.5, 150 mM NaCl. When necessary, the MIT domain was exchanged into a similar buffer with 300 mM NaCl, using PD-10 desalting columns (GE Healthcare, Uppsala, Sweden).

### 2.2 Preparation of vesicles

Lipids, 1-palmitoyl-2-oleoyl-sn-glycero-3-phosphatidic acid (POPA), 1-palmitoyl-2-oleoyl-sn-glycero-3-phosphatidylcholine (POPC), 1-palmitoyl-2-oleoyl-sn-glycero-3-phosphatidylethanolamine (POPE), and 1-palmitoyl-2-oleoyl-sn-glycero-3-phosphatidylglycerol (POPG), were obtained from Avanti Polar Lipids (Alabaster, AL). For circular dichroism, fluorescence and 1D NMR experiments, large unilamellar vesicles were prepared using extrusion as previously described [29,30]. Vesicles with zwitterionic lipids, POPC (100%) and POPE/POPC (40/60 mol%), and with negatively charged lipids, POPG/POPC (40/60 mol%) and POPA/POPC (10/90, 20/80, 30/70, 40/60 and 50/50 mol%), were produced. Lipids were dissolved in chloroform and the chloroform was evaporated overnight under nitrogen gas. The lipid mixtures were resuspended in 1 ml of the appropriate buffer for the experiment for 30 min by vortexing the samples. Next, each sample went through five cycles of freezing in liquid nitrogen and thawing in a water bath (∼60 °C). Finally, samples were extruded through a 100 nm polycarbonate membrane (Avanti Polar Lipids, Alabaster, AL). The size of the extruded vesicles was analyzed by dynamic light scattering (ALV-laser, Langen, Germany) and vesicles were found to have a radius of around 65 nm.

### 2.3 Circular dichroism spectroscopy

CD measurements were conducted on a Chirascan Circular Dichroism Spectrometer (Applied Photophysics, Leatherhead, UK) using a quartz cuvette with an optical path length of 0.05 mm (Hellma Analytics, Müllheim, Germany). Spectra were recorded at 25 °C from 190 nm to 260 nm and the bandwidth was 1 nm. For every sample, thirty spectra were recorded and averaged, and the background spectrum obtained in the absence of protein was subtracted. Three independent experiments were recorded for each measurement. The sample conditions were 2.5 mM lipids and 50 μM MIT domain in 50 mM phosphate buffer pH 7.5, 25 mM sodium fluoride, giving a protein/lipid ratio of 1:50. CD results are reported as mean residue ellipticities.

### 2.4 Fluorescence spectroscopy and acrylamide quenching

Fluorescence emission spectra were recorded with a Fluorolog fluorescence spectrometer (Horiba Scientific, Edison, NJ) at 25 °C using a quartz cuvette with an optical path length of 10 mm. The samples were excited at 295 nm and spectra were recorded from 310 nm to 400 nm in 1 or 0.5 nm steps with 2 nm excitation and emission slit widths. The sample conditions were 10 μM MIT domain and 500 μM lipids in 50 mM phosphate buffer pH 7.5 and 150 or 300 mM NaCl, giving a protein/lipid ratio of 1:50. Five repetitions were carried out for each sample. Quenching studies were performed using a Varian Cary Eclipse Fluorescence Spectrophotometer (Varian Inc., Mulgrave, Victoria, Australia). Aliquots from a 1 M stock solution of acrylamide (BioRad, Hercules, CA, USA) were titrated to the sample containing the MIT domain to concentrations ranging between 0 mM and 100 mM. Samples were mixed and incubated for 30 min before addition of acrylamide. Fluorescence was excited at 280 nm and scans were taken with a 5 nm excitation and emission slit width. The quenching constant (Ksv) was calculated with the Stern-Volmer equation:

*I*_*0*_/*I* = 1 + *K*_*SV*_ [*Q*]

Where I_0_ and I are the fluorescence intensities in the absence and presence of acrylamide, respectively, and [Q] is the concentration of acrylamide. The quenching constant was used to determine the degree of association of the MIT domain to vesicles of different composition. Three independent experiments were recorded for each measurement.

### 2.5 NMR spectroscopy

^1^H NMR spectra were acquired for samples containing 3.5 mM lipids, 28 μM MIT domain in 50 mM phosphate buffer pH 7.5, 150 mM NaCl, 5% D_2_O and 0.1 mM 4,4-dimethyl-4-silapentane-1-sulfonic acid for chemical shift referencing. Spectra were acquired at 25 °C on a Bruker 700 MHz spectrometer equipped with a cryo-probe. The 90° pulse length was 19 μs and 8000 transients were acquired.

## 3 Results

### 3.1 The MIT domain interacts specifically with phosphatidic acid

**Fig. 2:**
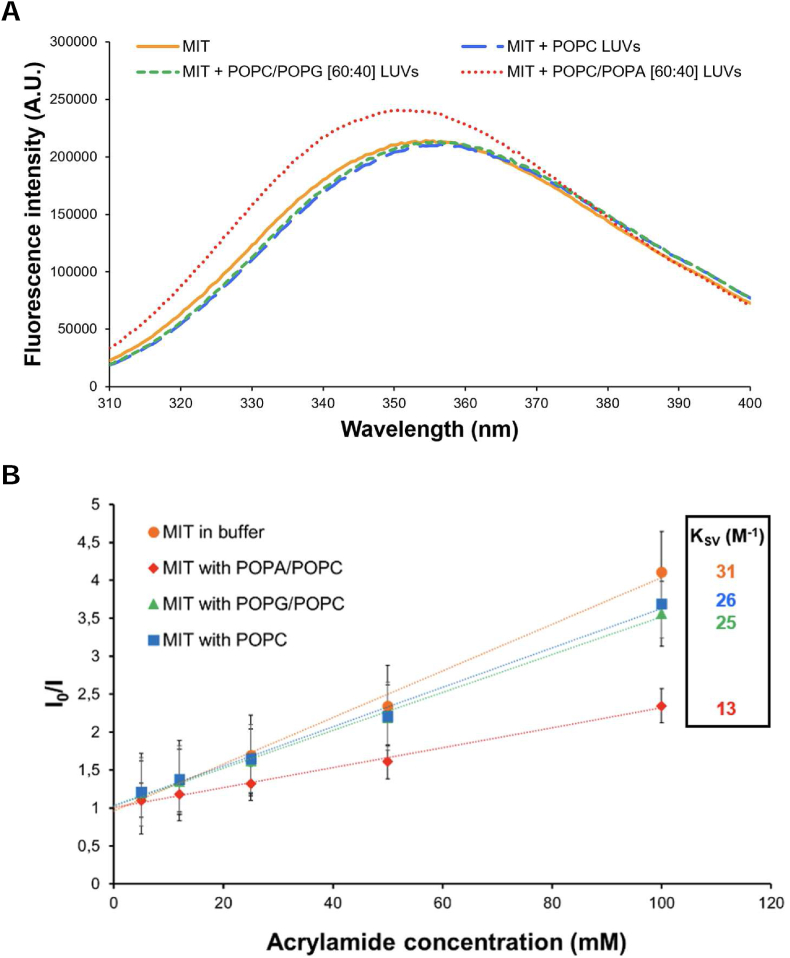
Tryptophan fluorescence data for the MIT domain. A: Spectra in 50 mM phosphate buffer and 150 mM NaCl (orange), with POPC/POPA (red), POPC/POPG (green) and POPC (blue) vesicles. B: Stern-Volmer plots and quenching constants for acrylamide quenching of MIT’s tryptophan fluorescence in the same solvents as in A. Standard deviations were obtained from three independent experiments. (For interpretation of the references to colour in this figure legend, the reader is referred to the Web version of this article.)

The MIT domain was expressed and purified as described previously [18] with the addition of size exclusion chromatography (SEC) to increase the purity of the protein (Fig. 1C and D). To test if the MIT domain in SmCHS 1 from *S. monoica* interacts specifically with certain lipids, fluorescence spectroscopy was used to assess both changes in spectral features, as well as to study the degree of insertion of the protein into a bilayer (Fig. 2). To produce a detectable change in the observed parameters, relatively high molecular ratios of PA are needed. The experiments thus probe the specificity towards a certain lipid rather than a true affinity. The MIT domain contains two intrinsic Trp residues, both located in the C-terminal helix 3 (Fig. 1) and the fluorescence spectrum is therefore a combination of the spectra for both Trp residues. Binding to large unilamellar vesicles containing 100% PC, 40% PA and 60% PC, or 40% PG and 60% PC, was studied. Significant blue-shifts in the maxima were observed as PA vesicles were added to the MIT domain, while only very modest shifts were observed for vesicles containing uncharged PC or anionic PG (Fig. 2A). Similarly, a significant increase in intensity was only observed in the presence of PA.

Quenching of intrinsic Trp fluorescence by the hydrophilic quencher acrylamide was also measured (Fig. 2B). The Stern-Volmer quenching constant for a free Trp residue in buffer is expected to be 35 M^−1^ [31], and here the quenching constant for the Trp residues of the MIT domain in buffer was observed to be only slightly less than that value (31 M^−1^), indicating an exposed location of the Trp side chains. Quenching by acrylamide became somewhat less effective as vesicles with either PC or PG were added to the protein (26 M^−1^ and 25 M^−1^ respectively), while a marked decrease in quenching efficiency was observed for vesicles containing PA (13 M^−1^). For Trp residues deeply immersed into a membrane the quenching constant can be close to 0 [31]. While we observe a marked decrease in the quenching constant in the presence of PA, the Trp residues are not completely buried, indicating a shallow binding to PA vesicles.

**Fig. 3:**
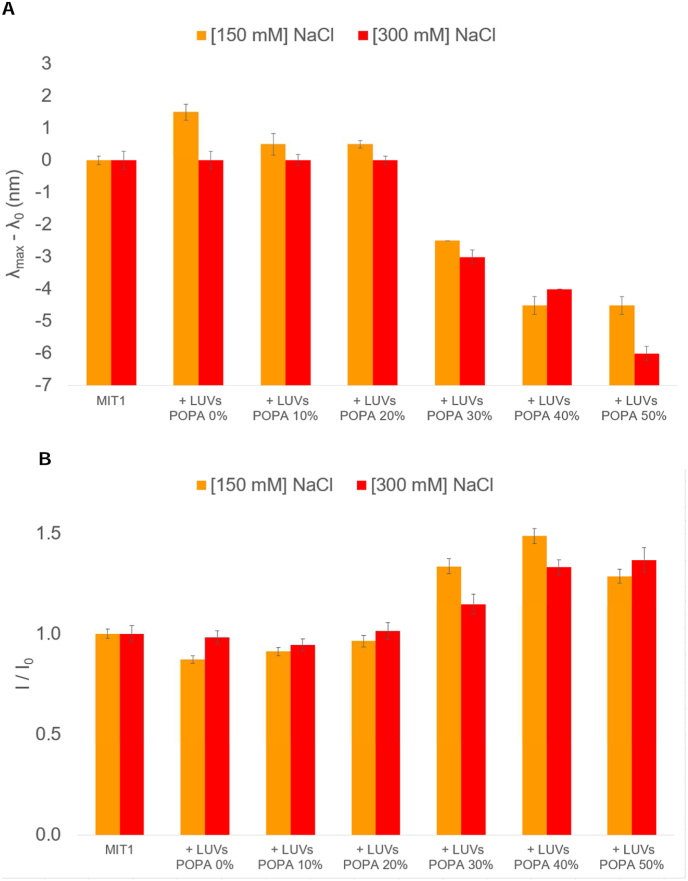
Tryptophan fluorescence data for MIT in the presence of vesicles containing increasing amounts of PA. Results for MIT in the presence of vesicles are shown relative to those in buffer. A: $\lambda_{max}-\lambda_{0}$ (the difference between the emission maximum, $\lambda_{max}$, and the emission maximum in buffer, $\lambda_{0}$), in the presence of either 150 mM NaCl (orange bars) or 300 mM NaCl (red bars). B: $I/I_{0}$ (the intensity of the emission maximum, I, relative to the emission maximum in buffer, $I_{0}$) in the presence of either 150 mM NaCl (orange bars) or 300 mM NaCl (red bars). Error bars signify errors calculated from ±2 standard deviations obtained from five measurements. (For interpretation of the references to colour in this figure legend, the reader is referred to the Web version of this article.)

To further test the binding specificity of the MIT domain for PA, LUVs with different amounts of PA were used, ranging from PC only to 50 mol% PA. These were used to monitor changes in fluorescence intensity and blue-shift (Fig. 3). In addition, to test if the interaction is purely electrostatic in nature, two concentrations of NaCl were used (150 and 300 mM) to monitor binding of the MIT domain to PA containing vesicles. No significant differences in emission maxima (Fig. 3A) or in peak intensities (Fig. 3B) were observed with varying amounts of NaCl, clearly indicating that the interaction cannot be screened by adding salt. Moreover, increasing the amount of PA in the vesicles had the effect of increasing fluorescence intensities as well as blue-shifts of emission maxima, irrespective of addition of NaCl. Taken together, the results support the conclusion that the MIT domain interacts preferentially with PA and that the interaction is not purely electrostatic in nature.

### 3.2 The structure of the MIT domain does not change upon lipid interaction

**Fig. 4:**
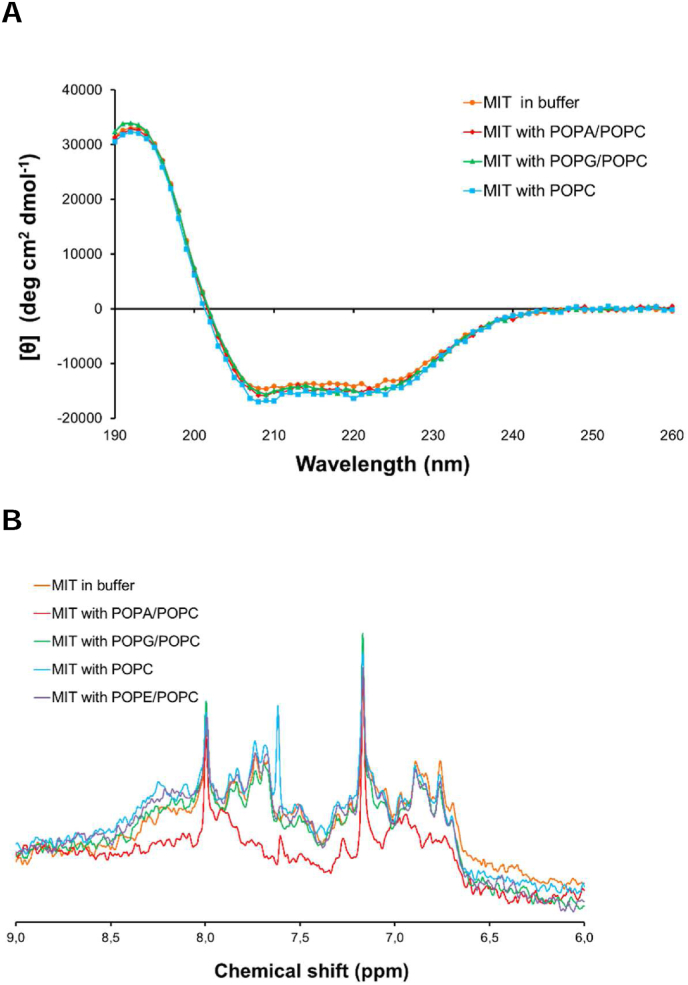
Structural data for MIT. A: CD spectra of MIT in buffer (orange) and in the presence of POPC/POPA (red), POPC/POPG (green) and POPC (blue). The CD spectra were recorded in triplicate. B: ^1^H NMR spectra of MIT in buffer (orange), and in the same buffer with POPC/POPA (red), POPC/POPG (green) and POPC (blue) and POPC/POPE vesicles (purple). (For interpretation of the references to colour in this figure legend, the reader is referred to the Web version of this article.)

To investigate if the structure of the MIT domain is affected by lipid interactions, far-UV CD spectroscopy was used to monitor changes in secondary structure (Fig. 4A). As expected, the CD spectrum displays characteristics of a highly helical protein in the absence of lipids. No significant changes in the CD spectrum were observed even in PA-containing vesicles, indicating that the MIT domain does not alter its structure upon lipid interactions.

To further test whether the MIT domain binds to vesicles and retains its structure, 1D ^1^H NMR spectra of the MIT domain in different vesicles were recorded. The amide region of the ^1^H NMR spectrum in buffer and in different vesicles is shown in Fig. 4B. No differences in the NMR spectrum of the MIT domain in PC- or PG-containing vesicles as compared to in buffer were observed. Addition of vesicles containing PA, however, led to a significant decrease in signal intensity due to strong line broadening, which is consistent with vesicle interaction. No effect of adding vesicles containing phosphatidylethanolamine lipids (PE) was observed, indicating that alterations in headgroup size is not sufficient to promote lipid interactions. In addition to causing broadening, the presence of PA shifted some peaks in the amide region. Since no significant changes were observed in the CD spectra, however, we conclude that the shift changes are due to changes in local environment, rather than a structural rearrangement. Our results demonstrate that the MIT domain interacts with PA in a way that does not involve any significant secondary structure rearrangements.

## 4 Discussion

The presence of specific lipids varies greatly during the life cycle of a cell, and the lipid composition is also specific for certain types of vesicles, important for cell signaling and trafficking [32]. The presence of MIT domains in chitin synthases has been suggested to be linked to membrane trafficking and therefore to lipid interactions [18]. In this work we have demonstrated that the MIT domain in chitin synthase 1 from *S. monoica* has a strong preference for interacting with phosphatidic acid over e.g. PG, which represents a generic negatively charged lipid. In previous work, we have established that the MIT domain interacts strongly with PA, while the protein does not interact with phosphatidylserine to a significant degree, although some degree of interaction with monophosphorylated phosphatidylinositols was observed [18]. These results suggest that a free phosphate group is necessary for lipid interaction, suggesting a specific mechanism for MIT-lipid interaction. Moreover, the interaction was, as observed also in this work, much stronger towards PA and it is therefore interesting to note that PA is a non-bilayer forming lipid. It has been suggested to be involved in membrane fusion and fission, due to its ability to alter the curvature of the membrane [23,33] and to give rise to strong electrostatic interactions [26,27].

The high ratios of PA needed to detect an interaction in the present study indicate that the MIT domain interacts with the membrane weakly. Another possibility is that the local concentration of PA varies in the membrane. Local concentrations of PA in *S. monoica* membranes are not known, but it is likely that it is present primarily in curved regions in the membrane, such as at the apex of the hyphae where CHS is found [8]. Although present in the plasma membrane in minor amounts, PA may form domains to which the MIT domain binds thus anchoring CHS to the membrane. Proteins involved in the delivery of CHS to the membrane in fungi have been identified [14], but microtubules and actin were not able to bind MIT domains from two *S. monoica* CHS, including the MIT domain studied here [18]. This supports the notion that direct interactions with lipids are a way to anchor the protein to the membrane. Only 15% of vesicles delivering CHS to the membrane fuse successfully with the plasma membrane [14], but the interactions between the MIT domain and PA could allow for a better rate of CHS insertion into the plasma membrane by increasing the residence time of the vesicle at the plasma membrane. In animal cells, successful exocytosis is defined by an extended tethering time (>10 s) at the plasma membrane [34].

The MIT domain contains ten basic amino acid residues, an attribute that has been suggested to be important for lipid interactions. MD simulations have suggested that binding to PA is achieved through a “hot-spot” containing several Arg residues (mostly so in helix 1 and helix 2) [22]. Here we see by fluorescence spectroscopy that the two Trp residues in helix 3 in the MIT domain interact with PA-containing vesicles. Inspection of the amino acid sequence shows that helix 3 has a marked amphipathic character with several basic amino acid residues (Lys48, Arg50, Lys56 and Lys58) and His residues (His55, His66 and His74) that are all located on the same side of the helix as the two Trp residues (Fig. 1).

Taken together, our data suggest that the interaction between the MIT domain and the lipid membrane are driven by specific properties of PA, such as strong electrostatic interactions between Lys residues and the phosphate headgroup as well as curvature stress induced by the small headgroup of PA. Our observations, specific binding to PA and shallow immersion of Trp residues, together with the structural properties of helix 3, exposed Lys, Arg, His and Trp residues, indicate that the binding of the MIT domain to PA is consistent with the "electrostatic/hydrogen bond switch model" [28]. In this model, basic residues in helix 3 form hydrogen bonds to PA’s phosphate headgroup decreasing its negative charge to −2. Hydrophobic interactions are facilitated by membrane packing defects due to the conical shape of PA and allow among others the Trp residues to form hydrophobic interactions that further enhance binding. In our model system, binding to PA is only observed for sufficiently high PA concentrations in the LUVs (>30 mol%). This might indicate that binding of the MIT domain can only be achieved when the density of membrane packing defects is sufficiently high and when a sufficiently high number of interactions between cationic residues and PA headgroups can be formed. Neither of the two effects alone seems to be sufficient for effective binding, as binding to PG and PE containing vesicles is either weak or non-existent.

## 5 Conclusion

We studied the interaction of the MIT domain of chitin synthase 1 with vesicles and found that it preferentially interacts with PA via electrostatic and hydrophobic interactions. The specific affinity of the MIT domain for PA suggests that this interaction serves a specific purpose *in vivo*. Interactions between the MIT domain and PA may be of relevance for the proper transport and localization of chitin synthase 1 to the plasma membrane, through fusion of transport vesicles to the membrane. Based on these and previous results we suggest a role for the MIT domain in insertion or in trafficking of the chitin synthase to the plasma membrane.

## Declaration of competing interest

The authors declare that there are no conflicts of interest.
